# Editorial: Therapeutic strategies and mechanisms for post-stroke emotional disorders

**DOI:** 10.3389/fpsyt.2023.1295940

**Published:** 2023-10-11

**Authors:** Huan Cai, Hao Zhang

**Affiliations:** ^1^Department of Rehabilitation Medicine, Zhongshan City People's Hospital, Zhongshan, Guangdong, China; ^2^Department of Neurology, Affiliated Hangzhou First People's Hospital, Zhejiang University School of Medicine, Hangzhou, Zhejiang, China

**Keywords:** stroke, emotional disorders, biomarker, treatment, mood

Stroke is a leading cause of disability and death worldwide (1) that impacts patients both physically and emotionally. The relationship between emotional disorders and stroke has received increasing attention in recent years. Emotional disorders, such as depression, anxiety, apathy, fatigue, and other neuropsychiatric symptoms, not only affect the onset of stroke (2) but are also related to the prognosis of stroke outcomes (3). Post-stroke emotional disorders have significant impacts on the activities of daily living, quality of life, rehabilitation, treatment compliance, and mortality of stroke survivors, which increase the burden on families and society. However, the neurobiological mechanisms remain largely unknown, which limits the efficiency of treatments. Therefore, in this Research Topic, we focused on the therapeutic, epidemiological, and genetics aspects of post-stroke emotional disorders, which may provide a closer insight into the neurobiological mechanisms and facilitate the treatment and prevention of emotional disorders after stroke.

Peripheral biomarkers have important value in the diagnosis of post-stroke emotional disorders and the development of new therapeutic approaches for them. Tao et al. explored the differential concentrations and potential clinical value of relevant blood markers in patients with vascular depression in a cross-sectional design. The results of their study indicate that hemoglobin (Hb), hypersensitive C-reactive protein (hs-CRP), cholinesterase (ChE), serum alkaline phosphatase (ALP), and high-density lipoprotein cholesterol (HDL-C) concentrations are potential blood markers of depression in cerebrovascular disease patients. Moreover, multivariate regression analysis showed an independent predictive value of ALP.

Approximately one-third of stroke survivors are affected by post-stroke depression (PSD), which is characterized by a depressive state after the onset of stroke (4). Vitamin D (VitD) is involved in many of the physiological activities of the brain. Sun et al. tried to establish the genetic link between VitD metabolic pathway genes (*VDR, CYP2R1, CYP24A1, CYP27B1*) and PSD in a Chinese population with ischemic stroke. In their study, they included 15 loci and obtained positive results, which showed that the G/G genotype in rs10877012 of the *CYP27B1* gene and the *VDR* rs11568820-rs1544410-rs2228570-rs7975232-rs731236 CCGAA haplotype may be associated with decreased risk of PSD. The results found in this study may help us to better understand the genetic background of PSD. However, the generalizability of these findings to other ethnic groups with a larger sample size is warranted.

Getting back to work after a stroke is an important rehabilitation goal for many working-age stroke survivors as it promotes one's quality of life, social integration, and emotional health. Besides physical and vocational rehabilitation, emotional factors (psychiatric disorders, fatigue, cognitive functioning) are also significant determinants of the return to paid work after a stroke. Chen et al. comprehensively reviewed randomized controlled trial literature on return-to-work programs for stroke patients, specifically examining components of mood and fatigue management upon the return to work. The results showed that emotion and fatigue are important predictors for stroke patients to return to work, and emotion and fatigue management can help young stroke survivors return.

Effective intervention for post-stroke emotional disorders is not enough, so it is urgent to investigate novel therapeutic strategies. Two studies in this Research Topic both focused on non-pharmacological interventions. The study conducted by Tham et al. evaluated the impact of a community-based cognitive intervention program called Train-Your-Brain (TYB) conducted in Singapore on depression, anxiety, and stress levels in stroke survivors and their caregivers. The participants of the TYB program form relationships within the group, which allows them to seek help from each other. This topic is significant as stroke survivors and their caregivers are at higher risk of experiencing mood disorders. The article offers preliminary evidence on the effectiveness of an intervention to support the wellbeing of stroke survivors. However, the caregivers experienced no significant improvements after the program. Another study focused on the role of social support in influencing PSD. Social support is defined as the exchange of physical or emotional resources between a provider and recipient with the aim of improving the recipient's wellbeing. Accordingly, Bi and Wang, employing meta-analysis, found that improving social support for stroke patients can help alleviate depression levels in individuals with stroke, which may bear useful clues in clinical practice for stroke caregivers.

In conclusion, the articles collected in this Research Topic included research on blood markers, genetic factors, and potential Non-pharmacological therapies for mood disorders among the stroke population. Recently, new promising therapeutic tools have been emerging, especially neuromodulation technology. Future research should continue to determine more specific biomarkers that may contribute to understanding post-stroke emotional disorders and examine the effects of interventions targeting these biomarkers for different emotional disorders.

## Author contributions

HC: Writing—original draft. HZ: Writing—review and editing.
